# Spatial normalization for voxel-based lesion symptom mapping: impact of registration approaches

**DOI:** 10.3389/fnins.2024.1296357

**Published:** 2024-01-17

**Authors:** Daniel Jühling, Deepthi Rajashekar, Bastian Cheng, Claus Christian Hilgetag, Nils Daniel Forkert, Rene Werner

**Affiliations:** ^1^Institute of Applied Medical Informatics, University Medical Center Hamburg-Eppendorf, Hamburg, Germany; ^2^Institute of Computational Neuroscience, University Medical Center Hamburg-Eppendorf, Hamburg, Germany; ^3^Department of Radiology, University of Calgary, Calgary, AB, Canada; ^4^Department of Neurology, University Medical Center Hamburg-Eppendorf, Hamburg, Germany; ^5^Center for Biomedical Artificial Intelligence (bAIome), University Medical Center Hamburg-Eppendorf, Hamburg, Germany

**Keywords:** VLSM, spatial normalization, image registration, stroke, neuroimaging

## Abstract

**Background:**

Voxel-based lesion symptom mapping (VLSM) assesses the relation of lesion location at a voxel level with a specific clinical or functional outcome measure at a population level. Spatial normalization, that is, mapping the patient images into an atlas coordinate system, is an essential pre-processing step of VLSM. However, no consensus exists on the optimal registration approach to compute the transformation nor are downstream effects on VLSM statistics explored. In this work, we evaluate four registration approaches commonly used in VLSM pipelines: affine (AR), nonlinear (NLR), nonlinear with cost function masking (CFM), and enantiomorphic registration (ENR). The evaluation is based on a standard VLSM scenario: the analysis of statistical relations of brain voxels and regions in imaging data acquired early after stroke onset with follow-up modified Rankin Scale (mRS) values.

**Materials and methods:**

Fluid-attenuated inversion recovery (FLAIR) MRI data from 122 acute ischemic stroke patients acquired between 2 and 3 days after stroke onset and corresponding lesion segmentations, and 30 days mRS values from a European multicenter stroke imaging study (I-KNOW) were available and used in this study. The relation of the voxel location with follow-up mRS was assessed by uni- as well as multi-variate statistical testing based on the lesion segmentations registered using the four different methods (AR, NLR, CFM, ENR; implementation based on the ANTs toolkit).

**Results:**

The brain areas evaluated as important for follow-up mRS were largely consistent across the registration approaches. However, NLR, CFM, and ENR led to distortions in the patient images after the corresponding nonlinear transformations were applied. In addition, local structures (for instance the lateral ventricles) and adjacent brain areas remained insufficiently aligned with corresponding atlas structures even after nonlinear registration.

**Conclusions:**

For VLSM study designs and imaging data similar to the present work, an additional benefit of nonlinear registration variants for spatial normalization seems questionable. Related distortions in the normalized images lead to uncertainties in the VLSM analyses and may offset the theoretical benefits of nonlinear registration.

## 1 Introduction

In population-level neuroimaging analysis, anatomical deviations of the individual human brains represent a source of uncertainty and a challenge to overcome. In practice, this challenge is tackled by the definition of a common coordinate system that allows a standardized interpretation of the burden of injury based on the image data of the individual patients. This step is commonly referred to as spatial normalization.

A typical example of the application of spatial normalization is in the context of voxel-based lesion-symptom mapping (VLSM). VLSM aims to assess the relation of lesions in specific brain regions with quantifiable clinical or functional deficits at a population level (Bates et al., 2003). For this purpose, the brain image data and the corresponding binary lesion maps of the patients are mapped into a common 3D coordinate system. The common coordinate system is usually defined by a population-averaged brain image, the atlas. Within the atlas space, a brain voxel is considered relevant in terms of its relation with the outcome measure if there is a statistically significant difference between the distribution of the outcome measures for the group of patients in whom the voxel is lesioned (that is, the voxel is part of the patient-specific lesion mask) and the group of patients in whom it is not. The overall result of the VLSM analysis is a map that represents the resulting value of the applied statistical test for each brain voxel of the atlas. Combined with a brain parcellation, the VLSM map allows further analysis of the potential importance of specific brain regions in relation to the outcome measure. Within this context, spatial normalization compensates for differences in location, size, and shape between brain images. Therefore, inaccurate spatial normalization introduces uncertainties in the computation of the VLSM maps.

**Figure 1:**
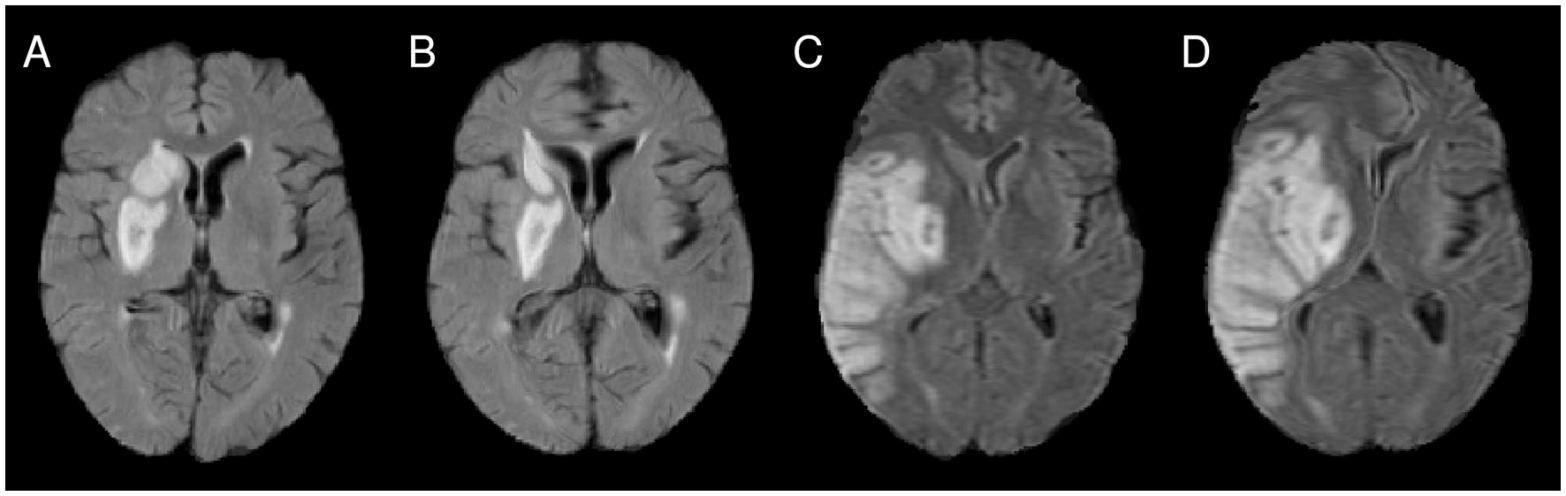
**(A)** Illustration of imprecise alignment of atlas and patient brain after affine registration (see, for instance, the midline and the lesioned periventricular tissue). **(B)** After nonlinear registration, the alignment was improved. However, for the patient shown in **(C, D)**, the nonlinear registration introduces a major midline shift **(D)**, compared to the alignment after affine registration **(C)**.

Spatial normalization is realized by registration of the patient images to the atlas image. To date, there is no consensus on the optimal approach, and different registration methods with varying complexity can be found in the VLSM literature. An often implemented basic approach is spatial normalization by *affine registration* (Chen and Herskovits, 2010; Cheng et al., 2014; Forkert et al., 2015), which allows for global translation, rotation, scaling, and shearing. Yet, affine registration is not able to compensate for regional, spatially restricted differences in brain anatomy (Figure 1A). Therefore, *nonlinear registration*, which allows for local deformations of the patient image, is also frequently used (Turken et al., 2008; Kalénine et al., 2010; Kielar et al., 2016; Tobyne et al., 2018; Leeuwis et al., 2019), usually applied as a refinement step after initial affine registration (Figure 1B).

The most common registration methods establish correspondence between the two images to be aligned (here: the patient brain image and the atlas image) by maximizing a similarity measure between the images, such as cross-correlation or mutual information. The similarity measure is evaluated for the entire image domain, and in the VLSM context typically for each voxel of the atlas space. This approach requires that the images to be registered represent the same anatomical structures. In turn, the presence of structures in one image without correspondence in the other leads to uncertainties and potential problems during registration. However, this is typically the case for data sets used within VLSM pipelines, for instance for stroke data sets: The lesions in the patient images (often represented as hypo- or hyperintense areas, depending on the imaging modality/sequences and stroke age) do not have a counterpart in the atlas image. While the effect on the global parameters computed in an affine registration is usually small, the effect of such non-correspondences on local transformation fields can be significant in nonlinear registration and the computed local deformation (Figures 1C, D). The severity of these effects further increases with lesion size (Andersen et al., 2010; Ripollés et al., 2012).

Two approaches are often used to counter related uncertainties in the VLSM context: *cost-function masking* (CFM) (Brett et al., 2001; Crinion et al., 2007; Andersen et al., 2010; Biesbroek et al., 2013; Pillay et al., 2014; Almairac et al., 2015; Pustina et al., 2017; Ghaleh et al., 2020) and *enantiomorphic registration* (Nachev et al., 2008; Yourganov et al., 2016; Salvalaggio et al., 2020; Hwang et al., 2021). CFM means that during nonlinear registration of the patient to the atlas image, the patient's lesion mask is used to define image areas that are not included in the computation of the similarity measure and related force and deformation fields. Thereby, the influence of the lesions on the registration result is assumed to be minimal. Enantiomorphic registration aims at minimizing the impact of the lesion during registration by replacing the lesion voxel intensities using contra-lesional voxel values. ENR therefore relies on the assumption that the brain consists of two homologous—essentially mirrored-symmetric—hemispheres.

In comparison, CFM may still lead to imprecise local normalization, especially for larger lesions, and error-prone interpolation of outer deformation fields inside the lesion mask. However, enantiomorphic registration will fail in the case of deviations from the underlying assumption, for instance in the presence of midline shifts in the patient image data caused by extensive water accumulation in the lesions often seen in stroke studies.

Thus, the applied registration approach and, therefore, the process of spatial normalization bears the risk of misrepresenting the size or shape of the patient's anatomical brain structures after mapping them into the atlas space. Consequences can range from small distortions to misplaced or “crushed” brain areas when compared to the original image data. In the worst case, the assumption of an anatomical correspondence between voxels and brain regions in the atlas and the patient image space is not fulfilled for patients of the considered population. However, this is the essential assumption for VLSM. The effects and implications of uncertainties introduced by the various normalization schemes available on VLSM maps have not yet been analyzed in detail. Therefore, the aim of this work was to evaluate the impact of the described commonly applied registration approaches, affine registration, (unmasked) nonlinear registration, CFM, and enantiomorphic registration, in the context of a typical VLSM scenario: the importance of (lesioned) brain voxels and regions with respect to modified Rankin scale (mRS) scores assessed 30 days after stroke (see, for instance, Cheng et al. 2014). The effects were analyzed for three different parcellation schemes and both univariate and state-of-the-art multivariate statistical testing.

## 2 Materials and methods

### 2.1 Imaging and clinical data

#### 2.1.1 Patient cohort

Magnetic resonance imaging (MRI) and clinical data from the European multicenter study I-KNOW were used in this work. Patients enrolled in I-KNOW underwent fluid-attenuated inversion recovery (FLAIR) MRI measurements between 2 to 3 days after showing first symptoms. Imaging was performed using a 1.5 T scanner. The in-plane resolution ranged from 0.43 × 0.43 mm^2^ to 0.94 × 0.93 mm^2^ and the between-plane resolution from 6.0 mm to 7.2 mm. Further imaging details can be found in Cheng et al. (2014). Data sets for this secondary study were made available after complete anonymization. Inclusion criteria for I-KNOW were first-ever stroke with ischemic stroke of the anterior circulation. Patients with bilateral lesions, injury to the brain stem or the cerebellum, and remote hemorrhagic transformations were excluded from this secondary analysis. For the image data of the remaining patients (*N* = 122; mean age 67.9 ± 12.2 years), skull stripping was performed using brain segmentation masks generated by a convolutional neural network with U-Net architecture trained on a subset of manually segmented images of 25 patients. If necessary, the brain masks were corrected manually. For all patients, binarized lesion maps were defined manually by an experienced observer (Rajashekar et al., 2022). Clinical outcome was measured by the modified Rankin scale (mRS) 30 days after stroke. The mRS is an ordinal outcome measure, consisting of seven grades ranging from 0 (no symptom) to 5 (severe disability) and 6 (death). Only patients without functional deficits before stroke (mRS 0 before stroke) were selected for this study.

#### 2.1.2 Atlas and parcellation schemes

The standard space into which the patient image data was mapped was defined by the GIN atlas (Lemaître et al., 2005). The GIN atlas is derived from T1-weighted brain scans from 662 healthy elderly patients (aged between 63 and 75 years) that matched the age distribution of the patients of the present study. The atlas image size is 256 × 256 × 128 voxel with an isotropic voxel size of 1 mm^3^. The Brain Extraction Tool (Smith, 2002) was used to generate a binary brain mask and to subtract all non-cerebral portions from the atlas image before image registration.

For region-based analysis of the VLSM maps for the different normalization approaches, three different parcellation schemes common in VLSM pipelines were used: The Harvard-Oxford cortical and the Harvard-Oxford subcortical atlas (Frazier et al., 2005; Desikan et al., 2006; Makris et al., 2006; Goldstein et al., 2007; Wu et al., 2015; Sihvonen et al., 2019) and the Johns Hopkins University (JHU) parcellation (Mori et al., 2005; Wakana et al., 2007; Hua et al., 2008; Knutson et al., 2014; Torso et al., 2015). The Harvard-Oxford subcortical atlas comprises 21 individual brain regions among which laterality (left/right hemisphere) is distinguished, while the Cortical Atlas consists of 48 brain regions without differentiation between hemispherically homologous regions. The JHU parcellation is a white matter atlas, comprising 20 white matter regions, distinguishing between left and right for homologous regions.

### 2.2 Registration approaches

Patient-to-atlas registration was performed using the Advanced Normalization Tools (ANTs) (Avants et al., 2009, 2011), an image processing toolkit widely used in neuroscientific and VLSM research (for instance in Kalénine et al., 2010; Pillay et al., 2014; Kielar et al., 2016; Pustina et al., 2017; Ghaleh et al., 2020; Hwang et al., 2021). The registration parameters were the default parameters detailed in Tustison and Avants (2013). As motivated in the introduction, four different registration approaches were applied: *affine registration* (AR), *nonlinear registration* (NLR), *nonlinear registration with cost-function masking* (CFM) using the binarized lesion maps as masks, and *enantiomorphic nonlinear registration* (ENR) using the lesion maps to define the voxel intensities to be replaced by the intensities of the corresponding voxels of the other hemisphere. The nonlinear registration approaches were applied to the warped FLAIR and lesion images after affine registration.

### 2.3 Voxel-based lesion symptom mapping

Voxel-level statistical differences of the mRS values 30 days after stroke between the two groups (patients with the voxel being lesioned vs. the other patients) were analyzed using non-parametric univariate as well as multivariate approach testing [univariate: Brunner-Munzel test; multivariate: SCCAN (Avants et al., 2014; Pustina et al., 2018), with sparseness optimization]. Both tests were applied employing the LESYMAP R package with a *p*-value threshold 0.05. Voxels with lesions in <10% of the patients were removed from the statistical analysis, and correction for multiple comparisons was performed by false discovery rate (FDR), representing a common VLSM setup. Thus, for each of the four normalization approaches (AR, NLR, CFM, ENR), two VLSM maps (univariate and multivariate analysis) were computed. For subsequent region-based analysis, a brain region from one of the three parcellation schemes was considered to be of importance to the mRS after 30 days if at least 1% of the region voxels exceeded the *p*-value threshold after correction for multiple comparisons.

### 2.4 Evaluation

The evaluation of the influence of the registration approaches on the normalization process and the VLSM results consisted of three parts: morphological analysis of the normalized FLAIR data; lesion volume evaluation before and after registration; and a comparison of the VLSM maps for the different parcellation schemes.

#### 2.4.1 Morphological analysis of FLAIR images

##### 2.4.1.1 Midline shifts

**Figure 2:**
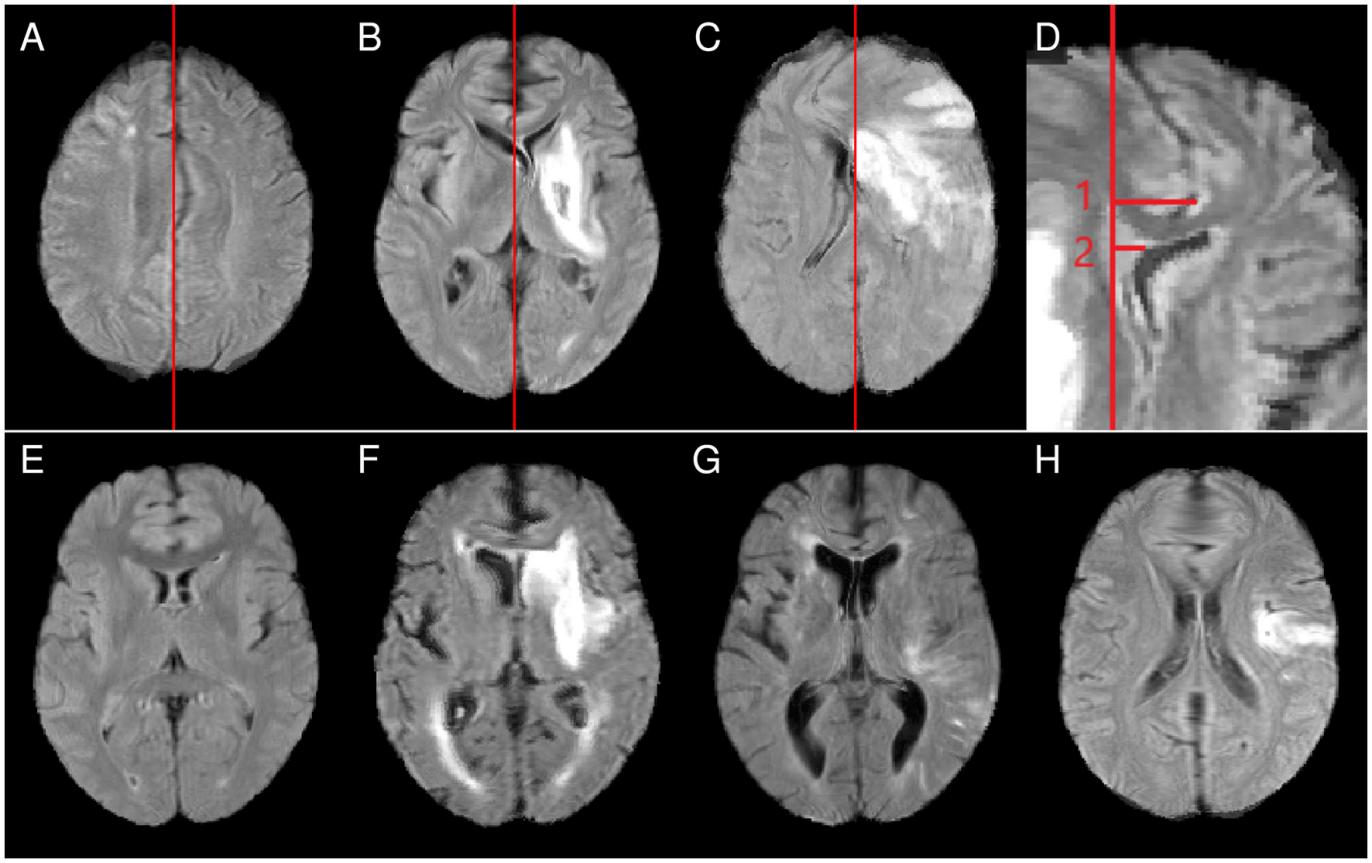
Top row: examples for midline shift scores [**(A)** score 1, midline shift limited to an area near the parietofrontal border, maintaining the original topology in other parts of the brain; **(B)** score 2, the thalami have shifted so far that involvement of other subcortical structures cannot be excluded; **(C)** score 3, wide-ranging topological distortions]. **(D)** Example measurements for the maximum midline shift (measurement 1) and the septum pellucidum shift (measurement 2). Bottom row: examples of abnormally small **(E)**, normal-sized **(F)**, and abnormally large **(G)** ventricles and a case showing a frontal blob **(H)**. All examples show cases *after* nonlinear normalization.

For LSM analyses, in principle, existing midline shifts in the original patient images should be compensated during a nonlinear normalization process. In practice, nonlinear registration can also introduce artificial midline shifts (that is, midline shifts that do not exist in the original image data), which can either be locally confined or more extended. Therefore, the presence of a midline shift before and after normalization was analyzed for all patients and normalization approaches and the severity of an existing midline shift was rated with a score from 1 to 3 (see examples in Figures 2A–C). A score of 1 indicated a focal deformation, that is, a midline shift in only a tightly circumscribed area that affected only a single brain structure. Regional deformations that affected not only a single brain structure but also nearby regions were rated with a score of 2. A score of 3 was assigned for global midline shifts and a deformation that also affected the topology of structures and regions further away from the deformation center.

In addition, the extent of post-registration midline shifts was quantitatively assessed as in clinical practice and described in Vande Vyvere et al. (2019). Therefore, the midline and the corresponding sagittal plane (coplanar with the falx cerebri) were identified in the atlas space. As illustrated in Figure 2D, from that plane, a line was perpendicularly drawn to the septum pellucidum. The length was defined as septum pellucidum (SP) shift (measurement 2 in Figure 2D). A second line measurement, again perpendicular to the defined plane and referred to as maximum midline (max) shift, ended at the point where the midline shift was at its largest extent (measurement 1 in Figure 2D). This measurement also included the interhemispheric fissure and the falx cerebri as potential endpoints, capturing all forms and localizations of post-registration midline shifts.

##### 2.4.1.2 Ventricle size and deformation

Nonlinear registration onto an atlas with normal-sized ventricles should ideally reduce the initial fraction of abnormally small and enlarged ventricles. Ventricles were therefore classified as abnormally small, normal-sized, or abnormally enlarged before and after normalization (see examples in Figures 2E–G). To gain further insight into local normalization effects, structures involved in abnormal ventricle deformation (anterior horns, posterior horns, center of the inner-brain ventricular system) and left-right asymmetries of the ventricular system were assessed.

##### 2.4.1.3 Frontal blobs

Nonlinear normalization was found to lead to spherical, anatomically not plausible deformations in the frontal brain region in some cases (Figure 2H). The frequency of frontal blobs was assessed for the different normalization approaches.

#### 2.4.2 Lesion volume analysis

Nonlinear registration can alter lesion appearance and size in an undesired way (“crushing” lesions, blurring lesions) to minimize the registration cost function. To evaluate this aspect, the lesion volume was assessed after applying the nonlinear registration approaches NLR, CFM, and ENR. As the affinely registered images formed the basis of the nonlinear registration step, the lesion volumes after NLR, CFM, and ENR were compared with the volumes after AR. Furthermore, possible correlations of lesion volumes and registration-relation lesion volume differences and correlations between lesion volumes and the findings of the morphological analysis (like SP and max midline shift) were analyzed.

#### 2.4.3 Impact of registration on VLSM maps

For each normalization approach and univariate as well as multivariate statistical testing, the corresponding statistical VLSM maps were computed. Similar to the lesion volume analysis, the resulting three-dimensional sets of significant voxels in the standard space were compared by size and their overlap quantified by the Dice coefficient. A potential shift of the sets toward the midline was analyzed by comparison of the center of gravity of the sets for the two hemispheres.

Furthermore, the overlaps of the sets of significant voxels with respect to mRS and the regions defined by the different parcellation schemes were evaluated. Normalization-related differences were analyzed and the plausibility of a contribution of the affected region was investigated under neuroanatomical, neurophysiological, and clinical aspects with regard to structure-function relationships.

## 3 Results

### 3.1 Morphological analyses before and after normalization

Five of the 122 patients (4%) showed an existing midline shift in the original and the affinely registered image data (only focal deformation for four patients; one patient with severity score 2). For all five cases, midline shifts were still present after nonlinear normalization and even worsened for three patients for all three nonlinear normalization approaches. Moreover, nonlinear normalization introduced midline shifts for patients without midline shifts in the original and affinely transformed images. For all nonlinear approaches, more than a quarter of the patients were affected (NLR: 30%; CFM: 25%; ENR: 30%). The median midline shift score of 2.0 (IQR: 1.0) as well as the median SP and maximum midline shifts [2.0 mm (5.0 mm); 6.0 mm (3.0 mm)] were lowest for CFM compared to NLR [score: 2.0 (1.5); SP shift: 4.0 mm (5.5 mm); max shift: 7.0 mm (7.0 mm)] and enantiomorphic registration [score: 3.0 (1.0); SP shift: 4.0 mm (7.0 mm); max shift: 10.0 mm (6.3 mm); *p* < 0.05 only for CFM and ENR score and max shift differences and NLR and ENR score differences, Wilcoxon rank sum test with Bonferroni correction]. Midline shifts mainly affected subcortical (NLR: 46.8% of the patients with midline shift; CFM: 40.5%; ENR: 57.1%) and frontal cortex areas (42.6%; 45.2%; 31.0%); parietal involvement was less frequent (NLR: 10.6%; CFM: 14.3%; ENR: 11.9%).

In the images before and after affine registration, the ventricles of 91/122 (74.6%) patients were rated as abnormally small or large (80 rated as enlarged, 11 as abnormally small). Nonlinear normalization reduced this fraction to 62.3% (NLR; images with enlarged ventricles: 64; abnormally small: 12), 68.9% (CFM; enlarged ventricles: 74; abnormally small: 10), and 58.2% (ENR;; enlarged ventricles: 62; abnormally small: 9), illustrating that all nonlinear registration approaches were not able to fully account for the corresponding individual differences. Abnormal ventricle deformation affected mainly the posterior horns (NLR: 94.7% of the patients with abnormally small or large ventricles affected; CFM: 91.7%; ENR: 98.6%) and centrally located compartments (NLR: 60.5%; CFM: 82.1%; ENR: 82.1%). Ventricle asymmetries were observed in approximately two-thirds of the patients (NLR: 61.5%; CFM: 67.2%; ENR: 68.9%).

Frontal blobs after nonlinear normalization occurred in 73.0% of the patients for NLR, 38.5% for ENR, and 9.0% for CFM. Affine normalization did not introduce frontal blobs.

### 3.2 Lesion volume analysis

Serving as the reference (see Section 2.4.2), the median lesion volume after affine normalization was 22.4 ml (range: 0.5–346.6 ml). NLR-based normalization resulted in a significant *increase* of the lesion volume (median volume: 23.5 ml; range: 0.6–373.7 ml; *p* < 0.001, Wilcoxon signed ranks test with Bonferroni correction); in contrast, CFM (median volume: 20.1 ml; range: 0.5–305.1 ml; *p*= 0.01) and ENR (median volume: 20.5 ml; range: 0.4–300.2 ml; *p*= 0.01) led to a *decrease* of the lesion volume compared to AR-based normalization. While there was a strong correlation between lesion volume and the observed absolute normalization-based lesion volume changes (in ml) for all nonlinear registration-based normalization approaches (Spearman correlation coefficients ρ between 0.74 for NLR and 0.85 for CFM), only a weak correlation was observed for the lesion volume and the relative lesion volume change (that is, the absolute lesion volume change divided by the lesion volume; ρ between −0.32 for ENR and 0.03 for CFM). Similarly, only a weak correlation was found between the extent of the normalization-induced midline shifts and the lesion volume of the patient (ρ between 0.3 and 0.4 for all normalization approaches and both SP and max shift). The lesion volumes of the patients in whom frontal blobs occurred after normalization did not differ significantly from those of the other patients (*p*> 0.10 for all normalization approaches). Similarly, lesion volumes in image data with abnormal ventricle size after normalization did not differ significantly from those with normal ventricle size (*p*> 0.27 for all normalization approaches).

### 3.3 Impact of registration on VLSM maps

**Figure 3:**
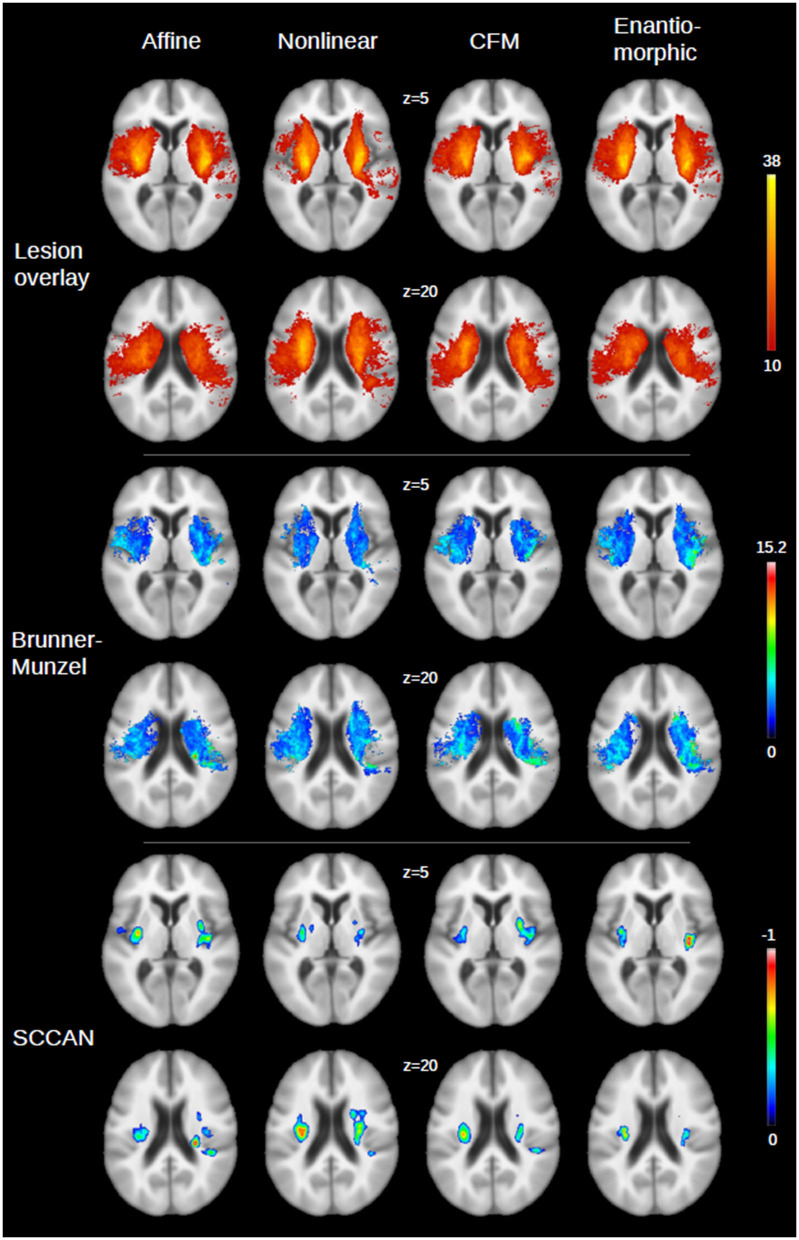
Illustration of the VLSM maps for the four registration approaches [affine, nonlinear, nonlinear with cost function masking (CFM) and enantiomorphic registration] for two axial slices (*z* = 5 and *z* = 20) of the GIN atlas. **(Top panel)** Overlay of the stroke lesions on days 2–3 after stroke onset from all patients (*N* = 122; color bar: number of overlapping lesions, with a lower threshold of 10, that is, the minimum number of lesions required to include a voxel in the statistical analysis). An overlay without lower threshold is shown in the Supplementary Figure S1. **(Middle)** Results for univariate statistical analysis (color bar: Brunner-Munzel *z*-score after multiple comparison correction). **(Bottom panel)** Corresponding results for multivariate analysis (SCCAN; color bar: normalized negative correlation with multiple comparison correction, as returned by LESYMAP).

The results of the voxel-wise statistical analyses of the relation of the FLAIR MRI lesions at days 2 to 3 after stroke onset and mRS after 30 days (that is, the VLSM maps) as well as the lesion overlay distribution are shown in Figure 3 for the different registration approaches applied for spatial normalization. For the univariate statistical analysis, the number of voxels that exceeded the significance threshold was not consistently larger or smaller after nonlinear registration-based normalization when compared to AR (NLR: 11% more significant voxels; CFM: +4%; ENR: −8%). For the multivariate statistical setting, the corresponding voxel set was smaller compared to AR in all nonlinear registration-based approaches (NLR: −21%; CFM: −53%; ENR: −23%). The Dice coefficients of the corresponding voxel sets were 77 and 75% (univariate statistical setting) and 48 and 50% (multivariate setting) for CFM and ENR when compared to AR. The overlap for NLR and AR was considerably smaller (58% for the univariate and 34% for the multivariate testing scenario). In line with this finding, for NLR, a clear shift of the significant voxel sets toward the midline was observed compared to AR (univariate statistical setting: shift of the center of gravity between 2 and 3 mm; multivariate: 6 mm). This shift was also present but less pronounced in ENR, but not in CFM.

The quantitative VLSM results for the different parcellation schemes and testing approaches (uni- and multivariate) are summarized in Table 1. In the following, regions were considered significantly related to and relevant for the mRS after 30 days only if >1% of their voxels in atlas space were evaluated to be significant. Table 1 focuses on anatomical regions that were evaluated to be relevant in at least one normalization and testing approach.

**Table 1:** Fraction of the voxels of the anatomical regions of the different parcellation schemes that were considered relevant for follow-up mRS outcome based on univariate (Brunner-Munzel) or multivariate (SCCAN) analysis.

**Brunner-Munzel**				**Label name**	**Region size**	**SCCAN**			
**AR**	**NLR**	**CFM**	**ENR**	**Harvard-Oxford cortical atlas**		**AR**	**NLR**	**CFM**	**ENR**
67.45	25.84	62.65	66.97	Insular cortex	13,909 vx	24.56	0.93	10.22	13.01
18.29	15.38	24.00	14.11	Frontal opercular cortex	1,821 vx	0.00	0.00	0.00	0.00
69.43	26.06	67.24	58.24	Central opercular cortex	8,200 vx	16.29	0.34	3.73	3.54
61.82	29.00	70.47	55.16	Parietal opercular cortex	3,410 vx	12.52	3.46	0.03	17.13
13.05	0.00	10.84	2.50	Planum polare	2,076 vx	0.10	0.00	0.00	0.00
71.02	1.26	85.54	51.07	Heschl's gyrus	1,349 vx	16.46	0.00	14.60	0.89
25.78	4.82	40.37	20.04	Planum temporale	2,056 vx	2.29	0.00	0.00	1.61
**AR**	**NLR**	**CFM**	**ENR**	**Harvard-Oxford subcortical atlas**		**AR**	**NLR**	**CFM**	**ENR**
24.88	10.19	8.33	25.10	Left caudate	3,662 vx	0.00	0.00	0.00	0.00
67.42	81.66	67.91	70.54	Left putamen	6,167 vx	10.54	15.73	1.54	23.67
10.45	71.65	10.65	25.94	Left pallidum	2,028 vx	0.00	0.00	0.00	0.00
0.05	0.10	0.00	5.10	Left amygdala	1,982 vx	0.00	0.00	0.00	0.00
4.64	0.00	0.01	2.68	Right lateral ventricle	7524 vx	0.00	0.00	0.00	0.00
0.14	3.37	0.00	1.57	Right thalamus	9,106 vx	0.00	0.00	0.00	0.00
22.84	2.08	10.76	31.26	Right caudate	3,800 vx	0.00	0.00	0.00	0.00
75.64	76.57	74.30	85.73	Right putamen	6,124 vx	18.01	16.77	19.55	28.09
47.94	89.72	45.54	49.55	Right pallidum	1,994 vx	0.65	16.70	0.70	1.81
0.00	1.08	0.00	0.61	Right Hippocampus	4,445 vx	0.00	0.00	0.00	0.00
3.70	3.61	0.75	7.00	Right amygdala	2,272 vx	0.00	0.00	0.00	0.00
**AR**	**NLR**	**CFM**	**ENR**	**JHU white matter atlas**		**AR**	**NLR**	**CFM**	**ENR**
14.81	21.56	15.00	18.70	Left ant. thalamic radiation	3,187 vx	0.00	0.00	0.00	0.13
12.51	27.44	6.05	11.53	Left corticospinal tract	1,735 vx	3.98	4.44	0.00	1.50
63.82	75.32	66.46	57.49	Left inf. fronto-occipital fasciculus	948 vx	7.28	1.27	1.58	0.84
0.00	3.39	0.09	0.00	Left inf. longitudinal fasciculus	1,062 vx	0.00	0.00	0.00	0.00
0.78	21.26	5.03	0.25	Left sup. longitudinal fasciculus	2,804 vx	0.00	0.93	0.00	0.00
86.90	82.14	78.57	92.86	Left uncinate fasciculus	84 vx	0.00	0.00	0.00	0.00
11.54	16.67	10.56	15.94	Right ant. thalamic radiation	3,570 vx	0.00	0.00	0.00	0.00
29.10	29.10	23.00	26.26	Right corticospinal tract	1,474 vx	9.84	8.55	2.44	12.69
53.05	53.55	53.71	47.03	Right inf. fronto-occ. fasciculus	1,212 vx	16.91	0.00	2.64	10.23
8.04	60.04	13.22	0.97	Right sup. longitudinal fasciculus	2,277 vx	0.04	0.61	0.00	0.00

Values are given in percentages. Only regions with a fraction of >1% in at least one normalization approach in uni- or multivariate statistical analysis are shown. Highlighted in blue: regions with more than 1% of their voxels showing a significant relation to mRS after 30 days.

#### 3.3.1 Harvard-Oxford cortical atlas

For univariate analysis, relevance for mRS after 30 days was ascribed to the same seven regions after AR-, CFM- and ENR-based normalization: the insular cortex, the frontal opercular cortex, central and parietal opercular cortex, the planum polare, the Heschl's gyrus, and the planum temporale. For NLR, the same regions except for the planum polare were found to be relevant, but the fraction of voxels evaluated to be significant differed from the corresponding AR, CFM, and ENR numbers for almost all regions (see Table 1 for details). Multivariate analysis consistently revealed importance for mRS after 30 days for the insular cortex and the central opercular cortex for AR, CFM, and ENR. The planum temporale was evaluated to be relevant in AR and ENR, while Heschl's gyrus was found as relevant for AR and CFM. After NLR-based normalization, only the parietal opercular cortex (similar to AR and ENR) was found to be important for mRS after 30 days.

#### 3.3.2 Harvard-Oxford subcortical atlas

Univariate statistical analysis revealed the left and right caudate nuclei, putamina, and pallidato to be of importance for mRS after 30 days for all registration approaches. After ENR, the left and right amygdalae, the right lateral ventricle, as well as the right thalamus were additionally evaluated to be relevant. The right lateral ventricle and the right amygdala were also of importance for AR. After NLR, the right hippocampus, the right amygdala, and the right thalamus revealed a fraction of voxels with *p* < 0.05. Multivariate statistical analysis showed consistently a relevant contribution to mRS after 30 days for the left and right putamina in all four registration approaches. Moreover, the right pallidum was found to be important after ENR- and NLR-based normalization.

#### 3.3.3 JHU parcellation

For the white matter atlas defined by the JHU parcellation and univariate statistical analysis, the left and right anterior thalamic radiations, the left and right corticospinal tracts, the left and right inferior fronto-occipital fasciculi, and the left uncinate fasciculus were evaluated to be important regions for mRS after 30 days for all four registration methods. For NLR, the left inferior longitudinal fasiculus as well as the left and right superior longitudinal fasciculi were also evaluated to be relevant. The latter two were also found to be relevant for CFM, and the right superior longitudinal fasciculus was found to be important in the affine normalization approach. In the multivariate analysis scenario, all four registration approaches consistently revealed a significant relation to mRS after 30 days only for the right corticospinal tract. In addition, AF, NLR, and ENR resulted in a relevant contribution by the left corticospinal tract. The left inferior fronto-occipital fasciculus was evaluated to be important for AR, NLR, and CFM, and the right inferior fronto-occipital fasciculus for AR, CFM, and ENR.

## 4 Discussion

The present study evaluated the impact of different but common spatial normalization approaches on VLSM maps using a standard registration toolkit (ANTs) with default parameterization (Avants et al., 2009, 2011). The VLSM use case was the analysis of the relation of FLAIR MRI lesions as present 2–3 days after stroke onset and functional outcome as measured by mRS after 30 days.

### 4.1 Morphological analysis

Registration aims to establish anatomical correspondence between two (or more) images. In VLSM context, anatomical correspondence is sought between an atlas image that defines a common coordinate system for subsequent statistical analyses on voxel level and the different patient images of a cohort. Because of anatomical variability of brain structures, it is often assumed that nonlinear registration is more appropriate than affine registration. However, the lesions in the patient images do not have a counterpart in the atlas image. While the effect on affine registration is usually small, the non-correspondences can significantly affect nonlinear registration and computed local deformations.

In the presented experiments, all nonlinear registration approaches (NLR, CFM, ENR; applied to refine the affine patient-to-atlas alignment) introduced artificial midline shifts in the warped patient images that were not present in the original FLAIR images. This affected more than 25% of the patients. In addition, so-called frontal blobs were consistently introduced by all nonlinear registration approaches.

Artificial midline shifts and frontal blobs were most pronounced after ENR and NLR. Standard nonlinear registration and the underlying cost function are affected by the presence of the (here: hyperintense) stroke lesion in the patient image, which has no correspondence in the atlas image. In enantiomorphic registration, this problem is addressed by replacing the intensity values within the lesion mask with intensity values of “corresponding” voxels of the contralateral hemisphere. Violations of the ENR symmetry assumption can again lead to strong local intensity variations and artificial intensity gradients, which lead to issues during cost function and force field computation. Both aspects could be explanations for the observed image distortions after ENR and NLR.

However, midline shifts and frontal blobs were also present after nonlinear registration with cost function masking (although with a lower frequency). This could be due to distortions in the original FLAIR images, which negatively affect nonlinear registration. In addition, from a methodical point of view, this could indicate insufficient regularization (that is, an insufficient transformation smoothness assumption) of the nonlinear registration. However, none of the nonlinear approaches was able to consistently normalize the ventricles, although an age-matched atlas was used: Ventricles that were deformed before normalization mainly continued to be either enlarged or small after nonlinear registration. This indicates a potential overregularization. Indeed, we were not able to find a better parameter setting than the default ANTs parameters used in this study to appropriately address this problem. Moreover, NLR led to a slight increase in the lesion volume compared to the affinely registered data, and CFM, while ENR resulted in a slight decrease. Lesion crushing was not observed. This finding contradicts the presence of drastic over- or underregularization.

In summary, based on the morphological analysis, an additional benefit of a nonlinear registration (after affine pre-alignment) seems questionable.

### 4.2 VLSM analysis

From a clinical perspective, the results presented in Section 3.3 are largely consistent with existing clinically oriented VLSM studies such as Cheng et al. (2014) and Ernst et al. (2018). Similarly, general differences between the results after uni- and multivariate analysis match previous findings (Avants et al., 2014; Ivanova et al., 2021). Since neither aspect was the main topic of this paper, the following discussion focuses on the differences in VLSM analysis results derived for the different normalization approaches.

#### 4.2.1 VLSM results: cortical atlas

Based on their location, several cortical regions were affected by the frontal blob phenomena (cingulate, paracingulate gyrus) and the midline shifts introduced by nonlinear normalization (cingulate, paracingulate gyrus, corpus callosum and associated structures, occipital and frontal poles, supracalcarine cortex). However, based on both uni- and multivariate statistical analysis, none of these structures had a significant association with the functional outcome, that is, mRS after 30 days, for any of the registration approaches. Thus, in the studied context of structure-function relationships in anterior circulation stroke, the distortions had no substantial effect on the VLSM maps and analysis results for the cortical structures.

**Figure 4:**
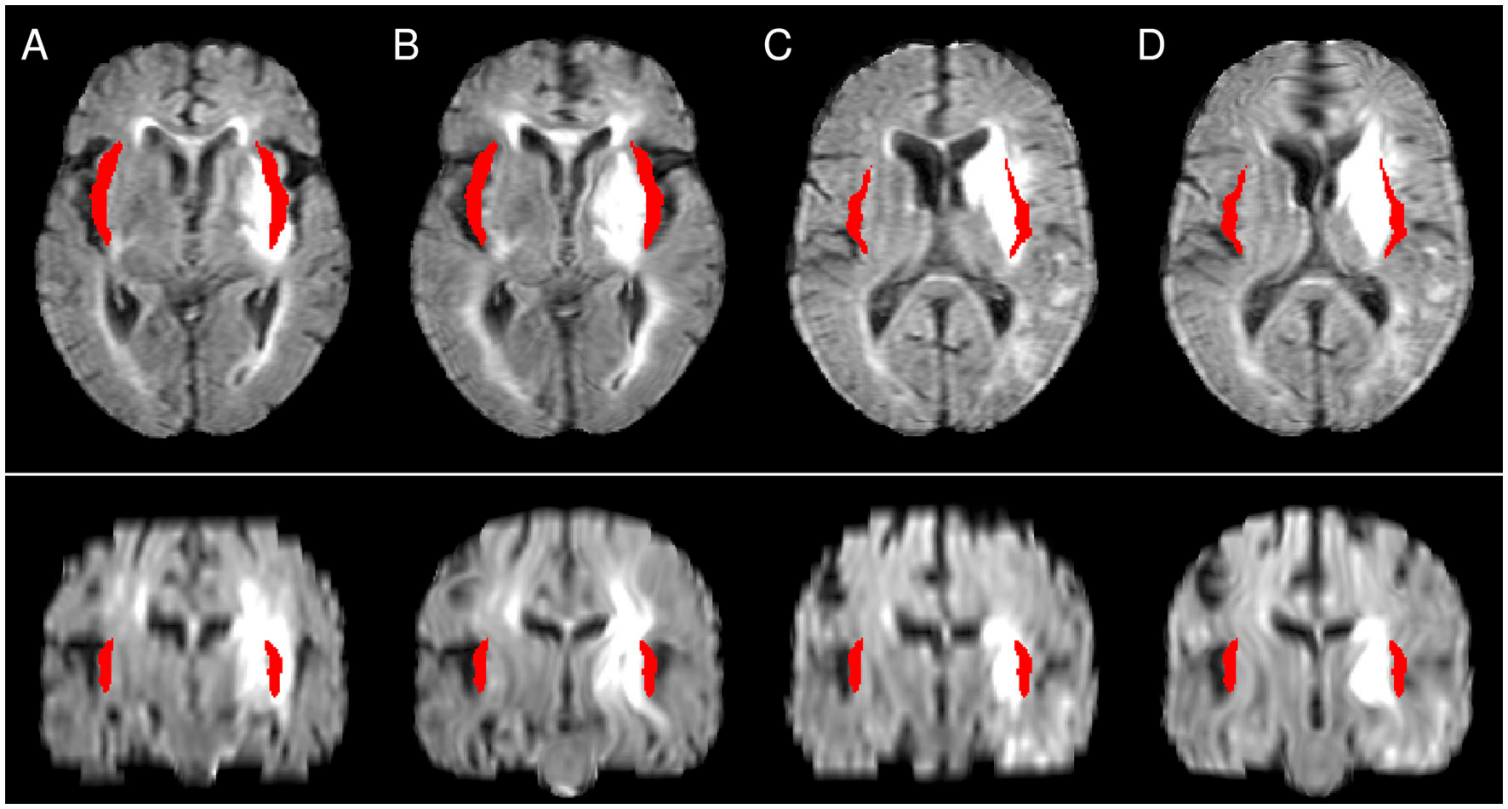
Illustration of the differences between CFM **(A, C)** and NLR **(B, D)**. The atlas mask for the insular cortex is shown in red. While in NLR peripheral structures such as the Sylvian fissure are moved into the mask area, the mask covers a lesioned area after CFM-based registration. **(A, B)** as well as **(C, D)** show registration results from the same patient.

Focusing on the structures of the Harvard-Oxford cortical atlas listed in Table 1, it is obvious that the fraction of significant voxels was significantly lower in NLR for most structures compared to the other registration approaches. The analysis of the set of significant voxels revealed that NLR shifted the structures and lesions closer toward the center, that is, the midline, than the other approaches. As a result, cortical structures were less likely to overlap with lesions, rendering corresponding results unreliable. In particular, for the insular cortex, which has been reported to significantly contribute to follow-up mRS in earlier studies (Cheng et al., 2014), no significant contribution was found after NLR-based normalization in the multivariate analysis setting. In detail, several examples were found, for which the Sylvian fissure was moved into the atlas area of the insular cortex, potentially replacing lesioned voxels (cf. Figure 4). Since the Sylvian fissure is a space filled with cerebrospinal fluid, it will not contribute to the functional outcome. In turn, the contribution of the atlas area “insular cortex” (and potentially also of other cortical structures like the different parts of the opercular cortex, the planum polare, the planum temporale and the Heschl's gyrus) may be underestimated for NLR-based normalization and the corresponding VLSM analysis.

#### 4.2.2 VLSM results: subcortical atlas

The nonlinear registration-associated frontal blob phenomena did not directly affect the structures of the Harvard-Oxford subcortical atlas. However, the introduced midline shifts affected them, especially the centrally located structures such as both thalami, the caudate nuclei, the lateral ventricles, and the nuclei accumbentes. In some severe cases, even peripherally located structures such as the lentiform nuclei were displaced in an implausible manner, with a potential impact on VLSM analysis results.

**Figure 5:**
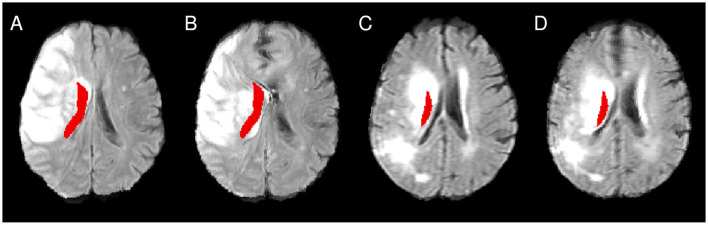
Illustration of the atlas right lateral ventricle mask being located inside the lesioned area after affine **(A, C)** and enantiomorphic registration **(B, D)**. **(A, B)** as well as **(C, D)** show registration results from the same patient.

In addition to the distortions, an insufficient normalization of the lateral ventricles potentially affects VLSM results for the ventricles themselves (which are parts of the atlas) and at least the directly adjacent subcortical structures. Table 1 shows that for affine and (to a smaller extent) enantiomorphic registration, a significant contribution to the prediction of the follow-up mRS is attributed to the right lateral ventricles (in the univariate setting). Since the ventricles are cavities filled with cerebrospinal fluid, this is not plausible and may indicate a spillover from adjacent regions. Visual inspection revealed that for a series of patient images after AR- and ENR-based normalization, the right lateral ventricle mask covered brain tissue, and partly lesioned tissue (Figure 5). In affine registration, the limited degrees of freedom do not allow local normalization of deformed ventricles, which explains this observation. For ENR, the increased frequency of midline shifts, which also affected the lateral ventricles (see above), could be an explanation of the values in the table. At this, the NLR and CFM results appear more plausible, although the fraction of patients with abnormally large or small ventricles after affine normalization was reduced by only 10%–20% (cf. Section 3.1).

In addition, the amygdalae were attributed with low fractions of significant voxels. However, the amygdalae are relatively small structures, and registration uncertainties and differences between normalization approaches for small structures can be expected to have a strong influence on the results when they affect the region, in particular when being located close to the ventricles. Corresponding results should therefore be interpreted with caution.

Apart from this, analysis of the VLSM results for the Harvard-Oxford subcortical atlas provided results consistent with clinical expectations. Consistently for all registration approaches and in both hemispheres, the univariate statistical analysis showed a significant association with the functional outcome for all regions that form part of the basal nuclei. However, in the multivariate analysis, the same degree of cross-hemispheric consistency among registration approaches was only observable for the putamina, and in AR and CFM, <1% of the voxels of the right pallidum exceeded the significance threshold. On visual inspection, these effects could not be directly attributed to obvious registration uncertainties or errors.

#### 4.2.3 VLSM results: white matter atlas

Among the white matter structures of the JHU atlas, only the forceps minor was at risk of being affected by the frontal blob distortions. Introduced midline shifts also affected the forceps minor and, in addition, the forceps major. Furthermore, VLSM results for structures directly adjacent to the lateral ventricles (left and right anterior thalamic radiations) could be influenced by severe midline shifts.

The anterior thalamic radiations were identified as relevant regions with respect to the follow-up mRS in the univariate analysis, but not in the multivariate analysis. Visual inspection of the normalized patient images did not provide a clear explanation of whether inadequate normalization of the lateral ventricles played a role in this finding. Furthermore, in the cost function masking approach and multivariate statistical analysis, the left corticospinal tract was not found to be a relevant region to follow-up mRS. Since the left corticospinal tract is one of the most important structures for motor function (Maraka et al., 2014) and thus for the mRS score, this seems implausible. In the same direction, Rajashekar et al. (2020) reported white matter tract integrity to be an important predictor of clinical outcome after ischemic stroke. However, lesion overlap in white matter tracts is not ideal for investigating the relation to functional outcomes, and it remains unclear whether this specific observation is due to the advantages or disadvantages of the normalization and testing procedures used in our study.

### 4.3 Further aspects

The morphological analysis and the analysis of the VLSM results for the different registration approaches showed that despite the nonlinear registration approaches led to distortions in the normalized images, the identified relevant brain structures for mRS 30 days after stroke onset were mostly consistent in both the uni- and the multivariate statistical testing setting. As discussed above, some (but not all) of the remaining differences could be explained by observed registration (and thus normalization) issues: underestimation of contributions by cortical structures after nonlinear registration, in particular NLR; overestimation of the contribution of, for instance, the lateral ventricles by AR due to insufficient normalization of the ventricles. However, the observed image distortions may have a much larger impact on different clinical outcome measures and scenarios, for instance, posterior circulation strokes.

In terms of additional limitations of the present study, we would like to note that we applied only one, although a very common one in VLSM context, registration toolkit with corresponding default parameters that were recommended by the developers. We ran several experiments trying to overcome the observed registration issues and image distortions, but, as discussed above, the trade-off between stronger regularization to avoid image distortions and less regularization to more appropriately normalize structures like the ventricles proved to be challenging. Although beyond the scope of the present study, it will nevertheless be interesting to study the effect of, for instance, more complex regularization schemes, different registration frameworks, and normalization approaches (Daws et al., 2022; Buch et al., 2023).

**Figure 6:**
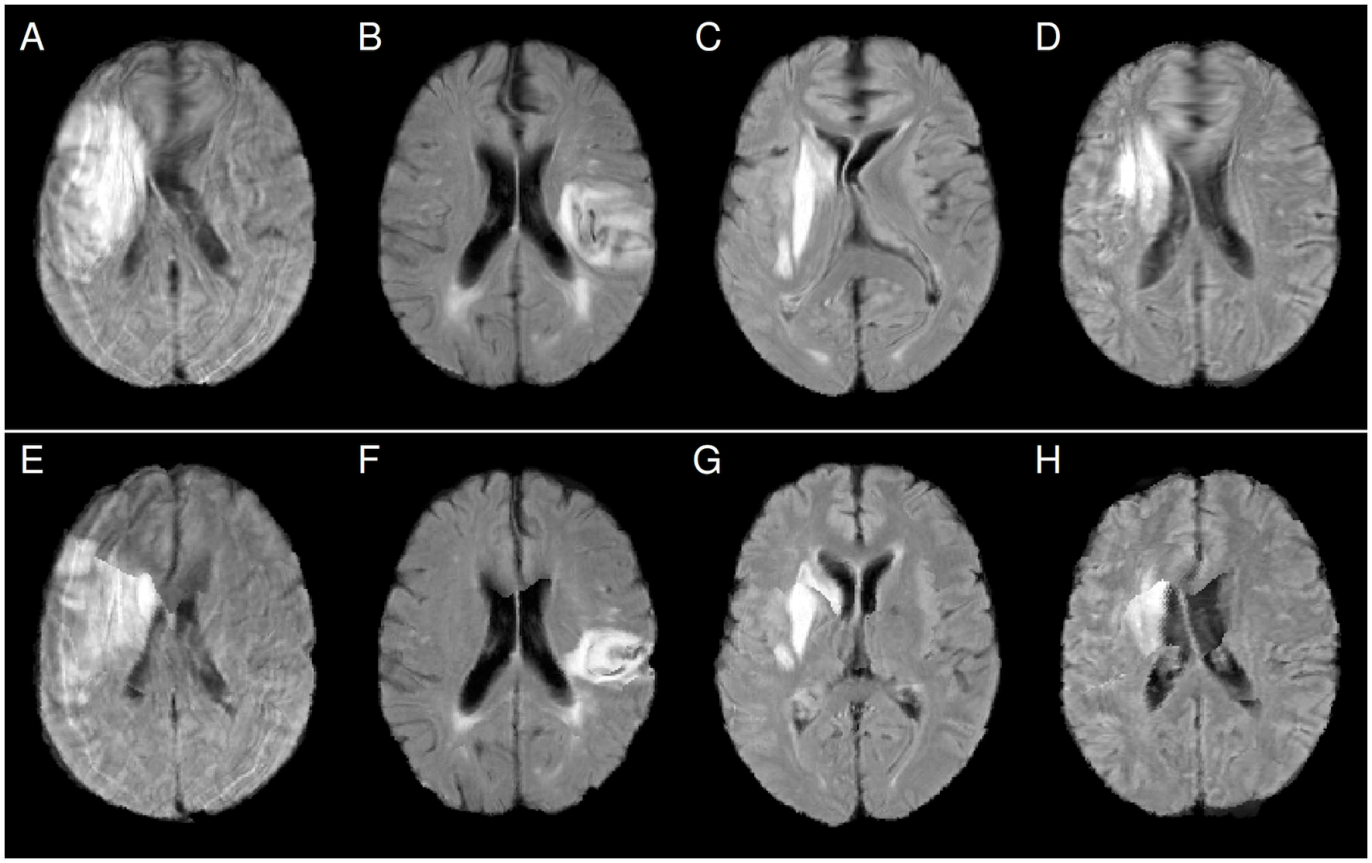
Comparison of FLAIR patient images after normalization using nonlinear FLAIR patient data to atlas registration **(panels A–D)** and nonlinear T1-weighted patient data to atlas registration **(panels E–H)**. Distortions like frontal blobs and midline shifts after normalization are clearly reduced after using the T1-weighted data.

Furthermore, our study was based on FLAIR MRI images with a relatively large slice thickness (6–7 mm). Better resolution of the patient images may also lead to better registration quality and less distortion of the normalized images. Moreover, T1-weighted MRI, T2-weighted MRI, or computed tomography data are often also available, suggesting the use of these images instead of the FLAIR data to compute nonlinear patient-atlas transformations that are potentially less distorted. The transformations can then be applied to normalize the FLAIR images. In the present study, for a limited number of patients, T1-weighted MRI datasets were available in addition to the FLAIR data. For those cases, we performed an affine registration between the FLAIR and the T1-weighted MRI data of the patient as well as affine and subsequent nonlinear registration between the T1-weighted patient data and the atlas image (which was computed from T1-weighted images so that the image modalities of patient and atlas images matched) with similar registration parameters as before. The resulting combined transformation was applied to the original FLAIR images. Figure 6 shows results for four patients who exhibited frontal blobs and midline shifts after NLR normalization without using the T1-weighted images (Figures 6A–D). Integrating the T1-weighted data into the pipeline leads to a clear reduction of the distortions (Figures E–H), although the issue of visible midline shifts after registration was not entirely resolved (Figures E, H). When T1-weighted or other additional images are not available or not for all patients (as in our study), the use of atlas images that better match the patients' image characteristics (such as imaging modality) could potentially also help to reduce the observed nonlinear registration problems.

Despite these limitations, the observed difficulties with nonlinear registration are known, making the derived VLSM results, or at least the envisioned advantages over affine registration and normalization, partially questionable. Moreover, although our study focused on ischemic stroke lesions, we hypothesize that the results are at least partially transferable to other lesion types such as hemorrhagic strokes, which can also be found in LSM context (Naidech et al., 2016; Moore et al., 2023). The missing lesion counterpart in the atlas image influences the nonlinear registration process and computed local deformations. The tested approaches (CFM and enantiomorphic registration) can reduce the influence, but residual effects remain, depending on further aspects like image quality and similarity of atlas and patient images.

Finally, it should be emphasized that the present analysis can only demonstrate that the VLSM results for the different registration approaches are *different*; despite plausibility considerations for some structures, it cannot provide evidence of which one is *more accurate* or reliable. This would require knowing the ground truth. In terms of future work, the results of this study encourage setting up further ground truth simulations that define the functional contributions of voxels and the way in which lesions impair their functions *a priori* (Mah et al., 2014). Such a setting will also allow us to study the obtained differences between uni- and multivariate testing in detail (a much larger extent of inferred significant voxels for the univariate setting, due to the known problem of incurring false positives in univariate lesion inferences) and the impact of the analysis parameters, which were chosen to be consistent with past analyses. This also includes the analysis of aspects like correction for potentially confounding factors such as lesion volume in the context of univariate testing setting [not considered in this technically-oriented study, but advantageous for a more reliable anatomical interpretation of the VLSM results (DeMarco and Turkeltaub, 2018)] and strategies for multiple comparison correction.

## 5 Conclusions

The reliability of voxel-based lesion symptom mapping results depends on the quality of the underlying spatial normalization, that is, the accuracy of the underlying registration of the patient image data and the atlas image. Nonlinear registration, and in particular variants that aim to reduce the undesirable impact of visible lesions during registration, are often considered better suited than affine registration. At least focusing on the present use case, that is the normalization of FLAIR MRI patient data using FLAIR patient data-to-atlas registration, the results illustrate that, contrary to intention, nonlinear registration approaches to spatial normalization may introduce new distortions for a substantial fraction of the patient images. In particular for standard nonlinear registration, the distortions and undesired effects of the lesions during registration have a substantial impact on VLSM maps and identification of regions significantly contributing to functional outcomes. The effects are less pronounced with more advanced spatial normalization methods such as using enantiomorphic registration and cost function masking, but they are still present, independent of the statistical testing approach.

The benefit of applying nonlinear registration-based normalization in VLSM context and using study designs and imaging data similar to ours is therefore questionable, at least, when applying out-of-the-box nonlinear registration-based solutions. If applied, nonlinear registration results have to be checked case-by-case, which appears hardly feasible for larger studies. In turn, when using affine registration alone, which does usually not require case-by-case control of the registration results, interpretation of VLSM results should also be done with caution, and a potentially insufficient local normalization needs to be taken into account.

## Data availability statement

The data analyzed in this study is subject to the following licenses/restrictions. Data has been obtained from a third party: the data analyzed in this study was obtained from the organizers of the I-KNOW study. Requests to access these datasets should be directed to BC, b.cheng@uke.de.

## Ethics statement

The studies involving humans were approved by Institutional Review Board of the University Medical Center Hamburg-Eppendorf. The studies were conducted in accordance with the local legislation and institutional requirements. The participants provided their written informed consent to participate in this study.

## Author contributions

DJ: Conceptualization, Data curation, Formal analysis, Investigation, Methodology, Validation, Visualization, Writing—original draft, Writing—review & editing. DR: Data curation, Formal analysis, Investigation, Methodology, Validation, Writing—review & editing. BC: Data curation, Resources, Validation, Writing—review & editing. CCH: Conceptualization, Funding acquisition, Validation, Writing—review & editing. NF: Conceptualization, Data curation, Methodology, Resources, Supervision, Validation, Writing—original draft, Writing—review & editing. RW: Conceptualization, Investigation, Methodology, Resources, Supervision, Validation, Writing—original draft, Writing—review & editing.
