# Supplementary material for: Spatial normalization for voxel-based lesion symptom mapping: impact of registration approaches
Source: Front Neurosci. 2024 Jan 17;18:1296357. doi: 10.3389/fnins.2024.1296357 (PMC10828036; doi:10.3389/fnins.2024.1296357)
Supplement: Supplementary file 1 [file Data_Sheet_1.PDF]

Supplementary Material

SUPPLEMENTARY FIGURES

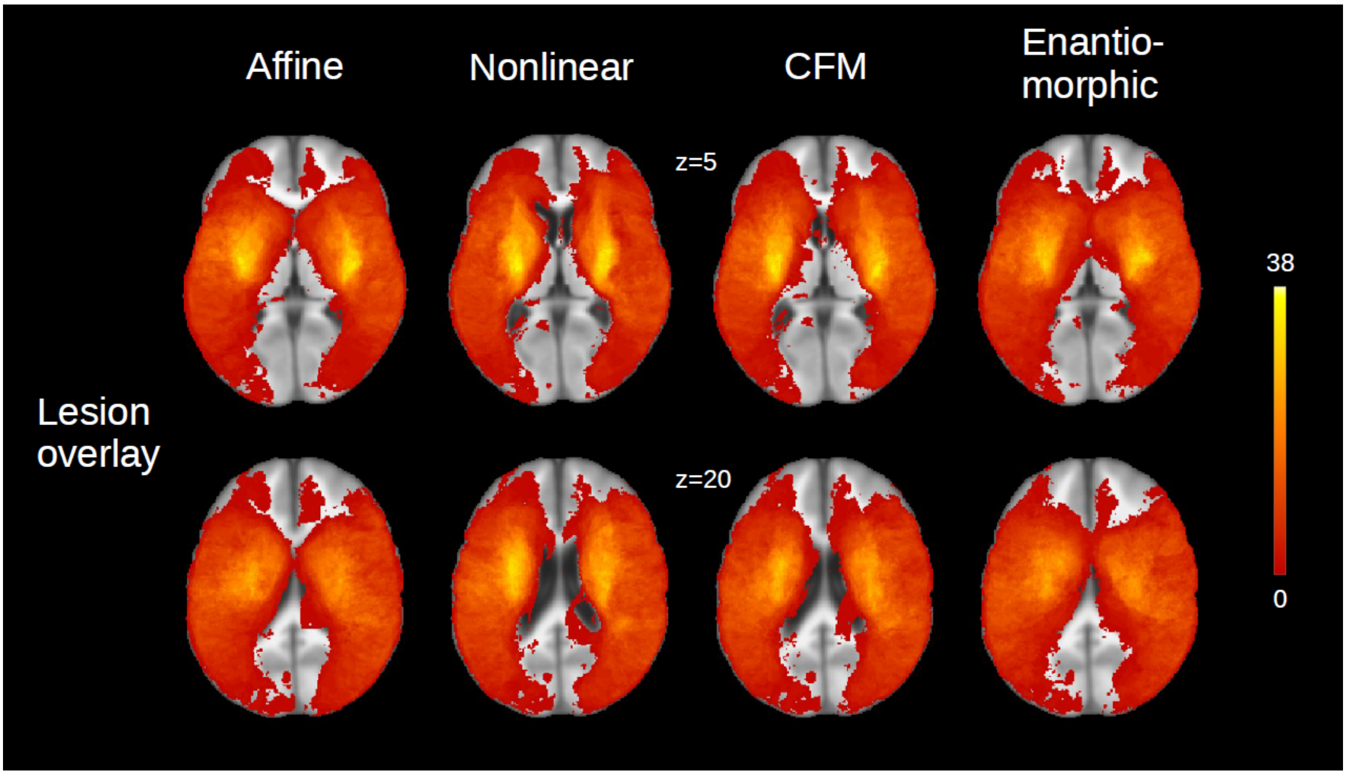

Figure S1. Overlay of the stroke lesions on days 2 to 3 after stroke onset from all patients (N=122; color bar: number of overlapping lesions). Similar to the top panel of Figure 3 of the main text, but without applying a lower threshold.
